# One Step Quantum Key Distribution Based on EPR Entanglement

**DOI:** 10.1038/srep28767

**Published:** 2016-06-30

**Authors:** Jian Li, Na Li, Lei-Lei Li, Tao Wang

**Affiliations:** 1 School of Computer Science, Beijing University of Posts and Telecommunications, Beijing 100876, China; 2 Hefei National Laboratory for Physical Sciences at Microscale and Department of Modern Physics, University of Science and Technology of China, Hefei, Anhui 230026, China; 3 JiLin Medical University, Jilin, 132013, China

## Abstract

A novel quantum key distribution protocol is presented, based on entanglement and dense coding and allowing asymptotically secure key distribution. Considering the storage time limit of quantum bits, a grouping quantum key distribution protocol is proposed, which overcomes the vulnerability of first protocol and improves the maneuverability. Moreover, a security analysis is given and a simple type of eavesdropper’s attack would introduce at least an error rate of 46.875%. Compared with the “Ping-pong” protocol involving two steps, the proposed protocol does not need to store the qubit and only involves one step.

The task of cryptograph is to ensure that only the legitimate users like Alice and Bob can read the secret message in the secure communication, which the unauthorized users like Eve cannot read. Researchers are dedicated to developing reliable and secure cryptographic protocols. With the rapid development of information technology and quantum physics^1^, quantum cryptography has become an important and attractive field. Quantum cryptography is based on the quantum mechanics, which is definitely different from the classical digital cryptographic system, and has much higher performance of security. With the rapid development of quantum mechanics in the past years, quantum information has been prosperous and fascinating.

Quantum mechanics offers some unique capabilities for the processing of the information, such as quantum computation and quantum communication. In the last decade, scientists have made dramatic progress in the field of quantum communication. The quantum key distribution (QKD), which task is to create a private key between two remote authorized users, is one of the most remarkable applications of quantum mechanics. Importantly, Gottesman, Lo, Lütkenhaus and Preskill (henceforth referred to as GLLP) proved the security of QKD when Alice’s and Bob’s devices are flawed, as is the case in practical implementations^2^. In addition, a device-independent QKD (DI-QKD) and measurement-device-independent QKD (MDI-QKD) was proposed. MDI-QKD protocol is fully practicable with current technology and attracted a lot of scientific attention from theoretical side^3^,^4^,^5^,^6^,^7^.

In 1984, C. H. Bennett and G. Brassard presented the pioneer quantum key distribution protocol, called BB84 protocol now^8^. This protocol has received lots of attention since it was come up. IBM and Montreal university first completed the experiment of quantum cryptography in 1989^9^, which verified the BB84 protocol from the aspect of experiment. In ref. 10, the communication distance extended to more than 1 km by use of polarized photons. Now the distance of key distribution can reach up to 200 km, and there are some other developments with quantum key distribution, such as refs 11, 12, 13, 14, 15, 16.

In recent years, a novel concept, quantum secure direct communication (QSDC) was put forward and studied by groups of researchers. It allows two remote parties to communicate directly without creating a private key in advance and using it to encrypt the secret message. Thus, the sender should confirm whether the channel is secure before he encodes his message on the quantum states because the message cannot be discarded, unlike that in QKD protocols. Many QSDC protocols have been proposed, including the protocols without using entanglement^17^,^18^,^19^, the protocols using entanglement^20^,^21^,^22^,^23^,^24^,^25^,^26^,^27^ and the two-way QSDC protocols^28^,^29^,^30^,^31^,^32^,^33^,^34^,^35^,^36^,^37^. The QSDC protocol can also be used in some special environments as first proposed by Boström *et al*.^38^ and Deng *et al*.^20^. In ref. 38, Boström and Felbinger presented a famous QSDC protocol which is called “Ping-pong” protocol. But researchers have found much vulnerability^39^,^40^,^41^,^42^ in the “Ping-pong” protocol.

A new quantum key distribution protocol was proposed in this paper, which based on entanglement and dense coding. However, in the new protocol, there is a serious problem that is the storage time limit during the actual operation. At present, the world record of quantum state storage time is only 3 ms at Hefei National Laboratory for Physical Sciences at Microscale and Department of Modern Physics. Considering the storage time limit, a grouping quantum key distribution protocol based on entanglement and dense coding, which does not need to store quantum states in process, is proposed. What’s more, the securities of these two protocols are analyzed.

## Results

### New QKD Protocol

Referring to the BB84 protocol and “Ping-pong” protocol, a new one step quantum key distribution protocol is proposed, which based on entanglement and dense coding. The entanglement mechanism is introduced to improve the security and the dense coding mechanism is introduced to increase the efficiency of transformation. For simplicity, we suppose that the new quantum key distribution protocol based on entanglement and dense coding in this paper is referred to as EQKD.

Now let us give an explicit process for EQKD. For easily understanding the process of EQKD, Table 1 shows an example.

1. Alice prepares a large enough number of classical bits N in sequence, and numbers the bits in the order Alice generates them.
2. Alice prepares enough EPR states in sequence based on the order of classical bit N and dense coding mechanism, and forms a series of particles S in order. Meanwhile, Alice remembers the entanglement states and the location information of every EPR states that the numbers of each EPR quantum bit in sequence S. Then Alice transfers the sequence S to Bob by quantum channel.
3. After Bob received the sequence S that Alice sent, Alice tells Bob the location information of every EPR states by classical channel.
4. Bob extracts every EPR states on the basis of location information of every EPR states, and then makes the Bell basis measurement accordingly.
5. Bob picks up a part of measurement results randomly as decoy photons and tells Alice the location information of the decoy photons. Alice tells Bob the original location information of the decoy photons. Then Bob compares his decoy photons with the original location information. If there is no eavesdropper, the error rate is lower than the threshold value, the quantum channel is deemed safe, and the generated raw key can be used. Otherwise, the generated raw key should be discarded, the communication is over.
6. Confirming the safety of channel, Bob and Alice negotiates about the remaining measurement results and performs the correction and privacy amplification, then obtains the finial key.

The quantum state 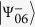 in Table 1 is referred to the 0^th^ bit and the 6^th^ bit in the classical bits forming 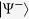 state. The four Bell states are shown as follows:
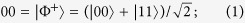

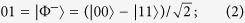

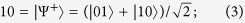

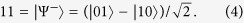


Suppose that the eavesdropper Eve uses the simplest intercept and retransmission scheme, which means that Eve intercepts the quantum bits that Alice transfers to Bob, and makes the Bell measurement to any two particles of all quantum bits randomly, then prepares quantum bits based on the measurement results and transfers to Bob. According to the Heisenberg’s uncertainty principle and no-clone theory, the Eve’s operation has to cause a certain error rate for Bob’s measurement. Because that Eve does not know the EPR location information of the quantum bits in EQKD protocol, the longer the quantum bits, the higher the error rate of Eve chooses the EPR location information randomly. Therefore, as long as the quantum sequence is long enough, the EQKD protocol is absolutely safe, and Eve cannot obtain any information. Then we can say that the EQKD protocol is asymptotically secure.

### Mini Protocol

One of the technical difficulties that have been unable to overcome is the ultrashort storage time of quantum state. All protocols that need to store quantum states in process have some limitations on the operability. The EQKD protocol which is proposed in this paper requires storing quantum states in process, so the operability of EQKD is low. On the basis of above facts, a grouping quantum key distribution protocol based on entanglement and dense coding, which does not need to store quantum states in process, is proposed in this paper. For simplicity, we suppose that the grouping quantum key distribution protocol based on entanglement and dense coding in this paper is referred to as MEQKD.

Every four bits of all classical bits is divided into a group in the MEQKD protocol and two EPR states is prepared for every group according to the entanglement and dense coding. Then the key information is transferred to Bob group by group. After receiving all groups in order, Bob obtains the final key.

Now let us give an explicit process for MEQKD. For easily understanding the process of MEQKD, Table 2 shows the process that every group is transferred to Bob.

1. Alice prepares a large enough number of classical bits N in sequence and every four bits of sequence N is divided into a group in order.
2. Alice picks up one group in order, and numbers the four bits into (1, 2, 3, 4). If all groups are took out, then go to the step (6); if all groups are not finished, then go to the step (3).
3. Alice prepares two EPR states in sequence based on the order of four bits of current group and dense coding mechanism, and forms a series of particles S. Meanwhile, Alice remembers the entanglement and the location information {(1, 2), (3, 4)} or {(1, 3), (2, 4)} of every EPR states. Then Alice transfers the sequence S to Bob by quantum channel.
4. After confirming that he received the sequence S, Bob randomly chooses one location information {(1, 2), (3, 4)} or {(1, 3), (2, 4)} to extract every EPR states, and then makes the Bell basis measurement accordingly.
5. After Bob finished measurement, Alice tells Bob the location information of every EPR states of current group by classical channel. If the location information that Bob chose is not right, then the generated key of current group is discarded; otherwise, Bob decodes the generated key of current group by dense coding mechanism, and adds them to the whole raw key. The process goes to step (2).
6. Bob picks up a part of whole raw key randomly as decoy photons and tells Alice the location information of the decoy photons. Alice tells Bob the original location information of the decoy photons. Then Bob compares his decoy photons with the original location information. If there is no eavesdropper, the error rate is lower than 46.875%, the quantum channel is deemed safe, and the generated raw key can be used. Otherwise, the generated raw key should be discarded, the communication is over.
7. Confirming the safety of channel, Bob and Alice negotiates about the remaining raw key and performs the correction and privacy amplification, then obtains the finial key.

The quantum state 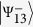 in Table 2 is referred to the 1^th^ bit and the 3^th^ bit in the classical bits forming 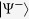 state.

The principle of the MEQKD protocol is similar to BB84, especially the random choose of EPR location information of every group, so the security of MEQKD protocol seems to be similar to that of BB84 protocol. In the MEQKD protocol, Bob chooses one of the EPR location information {(1, 2), (3, 4)} or {(1, 3), (2, 4)} to perform Bell measurement. The right probability of choosing EPR location information is 0.5, the probability of accurate measurement is 1. So the failure probability of choosing EPR location information is 0.5, according to the Heisenberg’s uncertainty principle, Bob will obtain a random result, and the probability of accurate measurement is 1/4 × 1/4 = 1/16 = 0.0625. Therefore, when there is no eavesdropper, the probability of right quantum bits which Bob can get is 1/2+1/2 × 1/16 = 17/32 = 0.53125^43^,^44^. Alice and Bob picks up one part of measurement results that chooses the same EPR location information and compares them via the classical channel, if the error rate is lower than the threshold value, the quantum channel can be seemed safe.

## Discussion

The family of individual attacks describes the most constrained attacks that have been studied. An important subfamily of individual attacks is the intercept-resend (IR) attacks, which Eve intercepts the quantum signal flying from Alice to Bob, performs a measurement on it, and, conditioned on the result she obtains, she prepares a new quantum signal that she sends to Bob. In the MEQKD protocol, this produces errors in the key Alice and Bob share. As Eve has no knowledge of EPR location information {(1, 2), (3, 4)} or {(1, 3), (2, 4)} sent by Alice is encoded in, she can only guess which qubit pairs to measure in, in the same way as Bob. If she chooses correctly, she measures the correct Bell state as sent by Alice, and resends the correct Bell state to Bob.

However, if she chooses incorrectly, the state she measures is random and the two qubits are not entangled, and the state sent to Bob cannot be the same as the state sent by Alice. Because there are two Bell states and every Bell state has four types (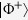, 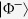, 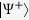, 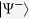), Bob gets the correct probability is 1/4 × 1/4 = 1/16. If Bob then measures this two Bell states in the same location Alice sent, he also gets a random result, and the correct probability is also1/4 × 1/4 = 1/16.

The Tables 3 and 4 show examples of this type of attack. In group 1 of Table 3, Eve selects right location, and Bob also selects the right location, the result is right. In group 2 of Table 3, Eve selects wrong location, and Bob select the right location, but the result of Bell states is wrong, so the keys are error. In group 3 of Table 4, Eve selects wrong location, and Bob select the right location, but one of the Bell state is right, the other Bell state is wrong, so the keys are error. In group 4 of Table 4, Eve selects wrong location, and Bob select the right location, but the result of Bell states is right, so the keys are right, probability of this situation is 1/16.

The probability Eve chooses the incorrect EPR location is 50% (assuming Alice chooses randomly), and if Bob measures this intercepted Bell states with the same location Alice sent he gets a random Bell state result, i.e., an incorrect result with probability of 15/16 = 93.75%. The probability two intercepted Bell state generate an error in the key string is then 50% × 93.75%  =  15/32 = 46.875%.

If Alice and Bob publicly compare *n* groups of their key bits (thus discarding them as key bits, as they are no longer secret) the probability they find disagreement and identify the presence of Eve is *P*_*d*_ = 1 − (17/32)^*n*^. So to detect an eavesdropper with probability of *P*_*d*_ = 0.999999999, Alice and Bob need to compare *n* = 33 key bits, while Alice and Bob need to compare *n* = 72 key bits in BB84 protocol.

Through the above analysis, the EQKD protocol is asymptotically secure, but the process that stores the quantum bits has some limitations on the operability. In the MEQKD protocol, the eavesdropper will introduce error rate of 46.875%.

Since Alice and Bob preserve only the part of the information that the same base they use when MEQKD protocol attacked by individual attacks, while in this part of the information, it is the probability of 1/2 to take the same base without introducing errors at this moment when Eve is eavesdropping; Simultaneously, it is the probability of 1/2 to take the different base and introducing errors with the probability of 15/32 at this moment, so the final result of the error rate is 15/32  =  46.875%. When Eve is eavesdropping, Eve gets 0 with the probability of 17/32 and gets 1 with the probability of 15/32 if Alice sends message 0; Similarly, Eve obtains 1 with the probability of 17/32 and obtains 0 with the probability of 15/32 if Alice sends message 1. Then, 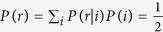, 
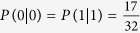
, 
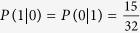
, thus we can get the mutual information:
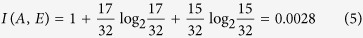


Although it does not meet the mutual information relations of privacy amplification, it will bring a greater error rate because that the mutual information is obtained by Eve’s eavesdropping in the process of all qubits transmitted. As long as setting a reasonable error rate upper limit (the upper limit is less than 46.875%), the communication processes that exceed the upper limit are canceled, and for the communication processes that the error rate do not exceed the upper limit, we are still able to ensure secure communication.

For MEQKD protocol, the amount of information that Eve obtained by coherent attack satisfies the following formula:
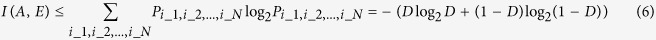
where D is QBER, *P*_*i*_1,*i*_2,…,*i*_*N*_ is the probability of the composition of the new quantum state when Eve’s detector obtained the quantum states *i*_1, *i*_2,…, *i*_*N* at the time of coherent measuring. And the mutual information of Alice and Bob is:
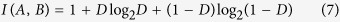


According to the conditions of error correction and privacy amplification *I*(*A*, *B*) > *I*(*A*, *E*), it must meets *I*(*A*, *B*) > *I*(*A*, *E*) at this time to achieve the purpose of secure communication, then the error rate D needs to satisfy the following formula:
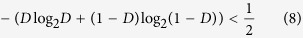
We can get the error rate upper limit D_0_ ≈ 11%.

In order to ensure security of MEQKD protocol for coherent attacks, we need to make the error rate D < 11%. With respect to individual attacks, the error rate upper limit is lower.

Compared with “Ping-pong” protocol, MEQKD has two merits, one is the MEQKD needs not to store the qubit, the other is that MEQKD only needs one step. The “Ping-pong” protocol requires two steps: the first step is Alice sends to Bob, another step is Bob sends it back to Alice. While MEQKD requires only one step, namely, Alice sends it to Bob, so called one step.

One of the localizations is that the preparation of Bell state used in the protocol is more difficult than the preparation of the single photon, while we believe that the problem will be solved with the advancement of technology.

The project is only a theoretical model and we do not consider the non-ideal conditions such as imperfect devices and noisy situations. In the further work, the experiment of this protocol will be made in Hefei National Laboratory for Physical Sciences at Microscale and Department of Modern Physics. The other QKD and QSDC protocol will be researched and the security of these protocols in noise channel and ideal channel will be analyzed.

## Additional Information

**How to cite this article**: Li, J. *et al*. One Step Quantum Key Distribution Based on EPR Entanglement. *Sci. Rep.* **6**, 28767; doi: 10.1038/srep28767 (2016).

## Figures and Tables

**Table 1:** The example of EQKD.

Number of classical bits	0	1	2	3	4	5	6	7	8	9
Classical bits that Alice prepares	1	1	0	0	0	1	1	0	1	0
EPR that Alice prepares	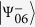	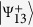	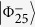	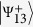	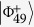	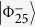	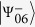	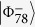	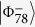	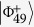
Measurement results that Bob makes	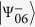	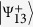	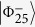	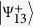	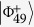	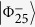	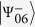	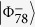	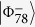	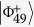
Detection particles	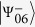		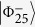			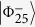	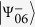			
Raw key that Bob obtains		1		0	0			0	1	0
Correction and privacy amplification		1		0				0	1	
Finial key	1001									

**Table 2:** The Example of the process that every group is transferred to Bob.

Number of every group classical bits	1	2	3	4
Classical bits that Alice prepares	1	0	1	1
EPR that Alice prepares	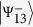	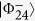	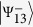	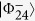
Measurement results that Bob makes	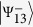	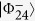	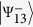	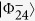
Every group raw key	1	0	1	1

**Table 3:** The example of intercept and resend attack(a).

	**Group 1**				**Group 2**			
Number of classical bits	1	2	3	4	1	2	3	4
Alice’s random bit	1	0	1	1	0	0	1	0
Alice sending Bell states	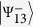	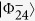	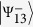	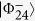	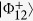	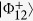	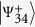	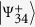
Eve random measuring basis	Bell(13)	Bell(24)	Bell(13)	Bell(24)	Bell(13)	Bell(24)	Bell(13)	Bell(24)
Eve selects right or wrong location	right				wrong			
Bell states Eve measures and sends	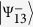	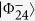	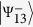	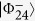	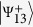	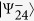	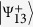	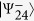
Bob random measuring basis	Bell(13)	Bell(24)	Bell(13)	Bell(24)	Bell(12)	Bell(12)	Bel(34)	Bel(34)
Bell states Bob measures	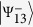	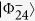	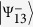	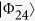	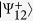	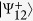	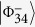	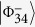
Public discussion of location	right				right			
Public discussion of states	right				wrong			
Share secret key	1	0	1	1	—	—	—	—
Errors in key	√	√	√	√	×	×	×	×

**Table 4:** The example of intercept and resend attack(b).

	**Group 3**				**Group 4**			
Number of classical bits	1	2	3	4	1	2	3	4
Alice’s random bit	0	0	1	0	0	0	1	0
Alice sending Bell states	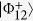	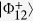	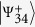	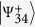	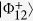	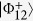	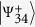	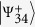
Eve random measuring basis	Bell(13)	Bell(24)	Bell(13)	Bell(24)	Bell(13)	Bell(24)	Bell(13)	Bell(24)
Eve selects right or wrong location	wrong				wrong			
Bell states Eve measures and sends	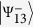	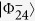	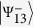	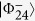	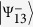	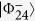	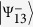	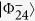
Bob random measuring basis	Bell(12)	Bell(12)	Bell(34)	Bell(34)	Bell(12)	Bell(12)	Bel(34)	Bel(34)
Bell states Bob measures	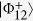	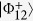	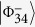	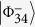	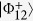	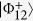	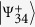	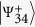
Public discussion of location	right				right			
Public discussion of states	right/wrong				right			
Share secret key	—	—	—	—	0	0	1	0
Errors in key	×	×	×	×	√	√	√	√

## References

[b1] Diffie W. & Hellman M. New directions in Cryptography. IEEE Trans. Inf. Theory 644–654 (1976).

[b2] Gottesman D., Lo H. K., Lütkenhaus N. & Preskill J. Security of quantum key distribution with imperfect devices. Quantum Inf. Comput. 4, 325 (2004).

[b3] Lo H. K., Curty M. & Qi B. Measurement device independent quantum key distribution. Phys. Rev. Lett. 108, 130503 (2012). 22540686 10.1103/PhysRevLett.108.130503

[b4] Xu F. H. . Discrete and continuous variables for measurement-device-independent quantum cryptography. *Nat. Photonics* 9(12), 772–773 (2015).

[b5] Zhu C. H., Xu F. H. & Pei C. X. W-state Analyzer and Multi-party Measurement-device-independent Quantum Key Distribution. Sci. Rep. 5, 17449 (2015). 26644289 10.1038/srep17449 PMC4672340

[b6] Zhao L. Y. . Measurement-device-independent quantum coin tossing. Phys. Rev. A. 92(6), 062327 (2015).

[b7] Cao Z., Zhou H. Y. & Ma X. F. Loss-tolerant measurement-device-independent quantum random number generation. New J. Phys. 17, 125011 (2015).

[b8] Bennett C. H. & Brassard G. Quantum cryptography: Public key distribution and coin tossing. *Proc. of IEEE Int. Conf. on Computers*, *Systems, and Signal Processing* [175–179] (IEEE, New York, 1984).

[b9] Bennett C. H. . Experimental Quantum Cryptography. J. Cryptol. 5(1), 3–28 (1992).

[b10] Muller A., Breguet J. & Gisin N. Experimental Demonstration of Quantum Cryptography Using Polarized Photons in Optical Fiber over More Than 1 Km. *Europhys. Lett*. 23(6), 383–388 (1993).

[b11] Dong C., Zhao S. H. & Sun Y. Measurement-device-independent quantum key distribution with q-plate. *Quantum Inf. Process*. 14(12), 4575–4584 (2015).

[b12] Guo Y., Lv G. L. & Zeng G. H. Balancing continuous-variable quantum key distribution with source-tunable linear optics cloning machine. *Quantum Inf. Process*. 14(11), 4323–4338 (2015).

[b13] Li H. W., Yin Z. Q. & Wang S. Randomness determines practical security of BB84 quantum key distribution. Sci. Rep . 5, 16200 (2015). 26552359 10.1038/srep16200 PMC4639782

[b14] Bahrani S., Razavi M. & Salehi J. A. Orthogonal Frequency-Division Multiplexed Quantum Key Distribution. J. Lightwave Technol. 33(23), 4687–4698 (2015).

[b15] Jasim O. K., Abbas S., El-Horbaty E. M. & Salem A. M. Quantum Key Distribution: Simulation and Characterizations. Proc. Comp. Sci . 65, 701–710 (2015).

[b16] Yang X. Q. . Trojan-horse attacks on counterfactual quantum key distribution. Phys. Lett. A., In Press, Accepted Manuscript *Available online 2*. (2015).

[b17] Deng F. G. & Long G. L. Secure direct communication with a quantum one-time pad. *Phys. Rev. A*. 69, 052319 (2004).

[b18] Cai Q. Y. & Li B. W. Deterministic secure communication without using entanglement. *Chinese Phys. Lett*. 21, 601–603 (2004).

[b19] Lucamarini M. & Mancini S. Secure deterministic communication without entanglement. Phys. Rev. Lett. 94, 140501 (2005). 15904054 10.1103/PhysRevLett.94.140501

[b20] Deng F. G., Long G. L. & Liu X. S. Two-step quantum direct communication protocol using the Einstein-Podolsky-Rosen pair block. *Phys. Rev. A*. 68, 042317 (2003).

[b21] Cai Q. Y. & Li B. W. Improving the capacity of the Boström- Felbinger protocol. *Phys. Rev. A*. 69, 054301 (2004).

[b22] Gao T., Yan F. L. & Wang Z. X. A simultaneous quantum secure direct communication scheme between the central party and other M parties. *Chinese Phys. Lett*. 22, 2473–2476 (2005).

[b23] Wang C., Deng F. G. & Long G. L. Multi-step quantum secure direct communication using multi-particle Green-Horne-Zeilinger state. *Opt. Commun*. 253, 15–20 (2005).

[b24] Li Y. H., Li X. L. & Nie L. P. Controlled Quantum Secure Direct Communication by Using a Five-Atom Cluster State in Cavity QED. Int. J. of Theor. Phys . 54(10), 3728–3732 (2015).

[b25] Wang L. L., Ma W. P. & Shen D. S. Efficient Bidirectional Quantum Secure Direct Communication with Single Photons in Both Polarization and Spatial-Mode Degrees of Freedom. Int. J. of Theor. Phys . 54(10), 3443–3453 (2015).

[b26] Li J., Pan Z. S. & Sun F. Q. Quantum Secure Direct Communication Based on Dense Coding and Detecting Eavesdropping with Four-Particle Genuine Entangled State. *Entropy* 17(10), 6743–6752 (2015).

[b27] Mi S. C., Wang T. J. & Jin G. S. High-Capacity Quantum Secure Direct Communication With Orbital Angular Momentum of Photons. *IEEE Photon. J*. 7(5), 1–8 (2015).

[b28] Nguyen B. A. Quantum dialogue. *Phys. Lett. A*. 328, 6–10 (2004).

[b29] Yadav P., Srikanth R. & Pathak A. Two-step orthogonal-state-based protocol of quantum secure direct communication with the help of order-rearrangement technique. *Quantum Inf. Process* 13(12), 2731–2743 (2014).

[b30] Ye T. Y. Quantum Secure Dialogue with Quantum Encryption. Commun. Theor. Phys. 62(3), 338–342 (2014).

[b31] Zou X. F. & Qiu D. W. Three-step semiquantum secure direct communication protocol. Sci. China Phys. Mech . 57(9), 1696–1702 (2014).

[b32] Xu S. J., Chen X. B. & Wang L. H. Two Quantum Direct Communication Protocols Based on Quantum Search Algorithm. Int. J. Theor. Phys. 54(7), 2436–2445 (2015).

[b33] Cao Z. W., Feng X. Y. & Peng J. Y. Quantum Secure Direct Communication Scheme in the Non-symmetric Channel with High Efficiency and Security. Int. J. Theor. Phys. 54(6), 1871–1877 (2015).

[b34] Li Y. B., Song T. T. & Huang W. Fault-Tolerant Quantum Secure Direct Communication Protocol Based On Decoherence-Free States. Int. J. Theor. Phys. 54(5), 1737–1737 (2015).

[b35] Lai H., Mehmet O. A. & Xiao J. H. Dynamic (2,3) Threshold Quantum Secret Sharing of Secure Direct Communication. *Commun. Theor. Phys*. 63(4), 459–465 (2015).

[b36] Li W. L., Chen J. B. & Wang X. L. Quantum Secure Direct Communication Achieved by Using Multi-Entanglement. Int. J. Theor. Phys. 54(1), 100–105 (2015).

[b37] Li X. H. Quantum secure direct communication. Acta Phys. Sinica . 64(16), 160307 (2015).

[b38] Kim B. & Timo F. Deterministic secure direct communication using entanglement. Phys. Rev. Lett. 89, 187902 (2002). 12398637 10.1103/PhysRevLett.89.187902

[b39] Wójcik A. Eavesdropping on the “Ping-pong” quantum communication protocol. Phys. Rev. Lett. 90(15), 157901 (2003). 12732070 10.1103/PhysRevLett.90.157901

[b40] Deng F. G. . Eavesdropping on the “Ping-pong” quantum communication protocol freely in a noise channel. *Chinese Phys. Lett*. 16, 277–281 (2007).

[b41] Cai Q. Y. The “Ping-pong” protocol can be attacked without eavesdropping. Phys. Rev. Lett. 91, 109801 (2003). 14525523 10.1103/PhysRevLett.91.109801

[b42] Zhang Z. J. & Man Z. X. The improved Boström-Felbinger protocol against attacks without eavesdropping. *Int. J. Quantum Inf*. 2, 521–527 (2004).

[b43] Zhao N. . Quantum key distribution secure threshold based on BB84 protocol. Acta Phys. Sinica 60(9), 090307 (2011).

[b44] Shor P. W. & Preskill J. Simple Proof of Security of the BB84 Quantum Key Distribution Protocol. Phys. Rev. Lett. 85(2), 441–444 (2000). 10991303 10.1103/PhysRevLett.85.441

