# Corrigendum: One Step Quantum Key Distribution Based on EPR Entanglement

**DOI:** 10.1038/srep46201

**Published:** 2017-04-25

**Authors:** Jian Li, Na Li, Lei-Lei Li, Tao Wang

Scientific Reports 6: Article number: 2876710.1038/srep28767; published online: 06 30 2016; updated: 04 25 2017

In the Article, we did not take into account the entanglement swapping that can be caused by eavesdropping efforts. Due to entanglement swapping, in Group 2 of Table 3 and Group 3 of Table 4, the initial states will never lead to a measurement outcome by Eve 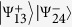 and 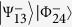. Similarly, if after Eve’s measurement Bob performs a measurement using an incorrect sequence, this will never lead to 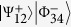 and 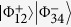. Therefore the outcomes for Group 2, Table 3, rows “Bell states Eve measures and sends” and “Bell states Bob measures”, and Group 3, Table 4, row “Bell states Bob measures”, need to be corrected. The correct versions of Table 3 and Table 4 are published below as Table 1 and Table 2 respectively.

Furthermore, these changes in the outcomes described in Table 3 and Table 4 lead to different results of the error rate evaluation. If Eve selects the wrong location to measure in the Bell basis, this will lead to entanglement swapping. The initial states 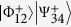 will become


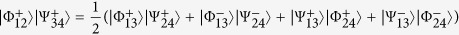


Suppose that if Eve’s measurement yields


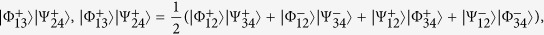


then Bob performing a measurement using a correct sequence after Eve’s measurement using an incorrect sequence will yield one of the following four possible results with equal probability: 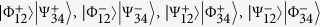.

Similarly, we can expand the other three possible outcomes of Eve’s measurement to


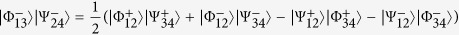



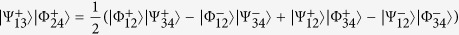



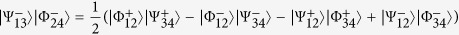


Due to this change, the error rate has to be corrected as follows.

On page 3 of the Article the paragraph:

“However, if she chooses incorrectly, the state she measures is random and the two qubits are not entangled, and the state sent to Bob cannot be the same as the state sent by Alice. Because there are two Bell states and every Bell state has four types 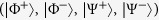, Bob gets the correct probability is 1/4 × 1/4 = 1/16. If Bob then measures this two Bell states in the same location Alice sent, he also gets a random result, and the correct probability is also 1/4 × 1/4 = 1/16”.

Should read

“However, Eve can still obtain the correct results with the probability of 1/4 if she chooses the wrong order, while she can obtain the wrong results with the probability of 3/4. So, the correct probability that Bob gets is 1/2 × 1/4 = 1/8. Therefore, the probability of the correct results that Bob will finally obtain is 1/2 × 1 + 1/2 × 1/4 = 5/8”.

On page 4 of the Article the paragraph:

Since Alice and Bob preserve only the part of the information that the same base they use when MEQKD protocol attacked by individual attacks, while in this part of the information, it is the probability of 1/2 to take the same base without introducing errors at this moment when Eve is eavesdropping; Simultaneously, it is the probability of 1/2 to take the different base and introducing errors with the probability of 15/32 at this moment, so the final result of the error rate is 15/32 = 46.875%. When Eve is eavesdropping, Eve gets 0 with the probability of 17/32 and gets 1 with the probability of 15/32 if Alice sends message 0; Similarly, Eve obtains 1 with the probability of 17/32 and obtains 0 with the probability of 15/32 if Alice sends message 1. Then, 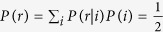, 
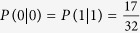
, 
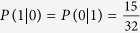
, thus we can get the mutual information:


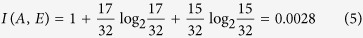


Should read

Alice and Bob preserve only the part of the information that they use the same base when MEQKD protocol is attacked by individual attacks. In this part of the information, there is a probability of ½ of taking the same base without introducing errors when Eve is eavesdropping. Simultaneously, there is a probability of 1/2 of taking the different base, and the probability of introducing errors is 3/8 at this time. So the final result of the error rate is 3/8 = 37.5%. When Eve is eavesdropping, Eve gets 0 with the probability of 5/8 and gets 1 with the probability of 3/8 if Alice sends the message 0. Similarly, Eve gets 1 with the probability of 5/8 and gets 0 with the probability of 3/8 if Alice sends the message 1. Then, 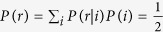, 
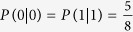
, 
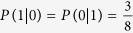
. Thus, we can get the mutual information:


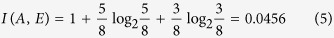


Note that the security for the QKD scheme proposed in the Article has not been proven yet.

## Figures and Tables

**Table 1:** The example of intercept and resend attack (a).

	Group 1				Group 2			
Number of classical bits	1	2	3	4	1	2	3	4
Alice’s random bit	1	0	1	1	0	0	1	0
Alice sending Bell states	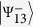	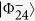	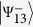	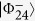	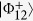	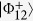	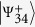	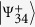
Eve random measuring basis	Bell(13)	Bell(24)	Bell(13)	Bell(24)	Bell(13)	Bell(24)	Bell(13)	Bell(24)
Eve selects right or wrong location	right				wrong			
Bell states Eve measures and sends	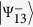	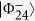	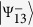	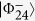	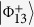	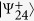	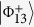	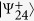
Bob random measuring basis	Bell(13)	Bell(24)	Bell(13)	Bell(24)	Bell(12)	Bell(12)	Bel(34)	Bel(34)
Bell states Bob measures	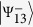	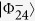	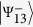	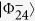	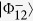	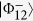	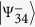	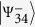
Public discussion of location	right				right			
Public discussion of states	right				wrong			
Share secret key	1	0	1	1	—	—	—	—
Errors in key	√	√	√	√	×	×	×	×

**Table 2:** The example of intercept and resend attack (b).

	Group 3				Group 4			
Number of classical bits	1	2	3	4	1	2	3	4
Alice’s random bit	0	0	1	0	0	0	1	0
Alice sending Bell states	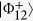	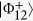	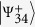	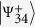	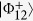	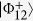	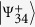	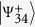
Eve random measuring basis	Bell(13)	Bell(24)	Bell(13)	Bell(24)	Bell(13)	Bell(24)	Bell(13)	Bell(24)
Eve selects right or wrong location	wrong				wrong			
Bell states Eve measures and sends	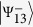	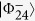	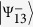	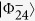	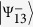	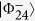	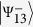	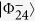
Bob random measuring basis	Bell(12)	Bell(12)	Bell(34)	Bell(34)	Bell(12)	Bell(12)	Bel(34)	Bel(34)
Bell states Bob measures	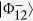	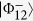	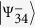	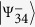	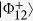	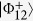	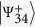	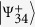
Public discussion of location	right				right			
Public discussion of states	right/wrong				right			
Share secret key	—	—	—	—	0	0	1	0
Errors in key	×	×	×	×	√	√	√	√

