# Corrigendum: One Step Quantum Key Distribution Based on EPR Entanglement

**DOI:** 10.1038/srep47004

**Published:** 2018-07-18

**Authors:** Jian Li, Na Li, Lei-Lei Li, Tao Wang

Scientific Reports 6: Article number: 2876710.1038/srep28767; published online: 06 30 2016; updated: 07 18 2018

The Corrigendum published 25 April 2017 did not explain that the issue of entanglement swapping had been raised previously by Pathak and Thapliyal^1^.
